# Voltage-Dependent Ion Channels in Vascular Endothelial Cells: An Unexpected Signaling Pathway in Non-Excitable Cells

**DOI:** 10.3390/biomedicines14071418

**Published:** 2026-06-23

**Authors:** Francesco Moccia, Teresa Soda

**Affiliations:** 1 Department of Medicine and Health Sciences “Vincenzo Tiberio”, University of Molise, 86100 Campobasso, Italy; 2 Department of Health Sciences, University of Magna Graecia, 88100 Catanzaro, Italy; teresa.soda@unicz.it

**Keywords:** vascular endothelial cells, voltage-gated sodium channels, voltage-gated calcium channels, voltage-gated potassium channels, angiogenesis, vasorelaxation, inflammation

## Abstract

Voltage-gated ion channels (VGICs) are traditionally associated with electrically excitable cells; however, increasing evidence indicates that they are also expressed in non-excitable cells, including vascular endothelial cells. This review aims to summarize the current knowledge on the expression, regulation, and functional role of VGICs in the vascular endothelium, and to highlight their potential contribution to endothelial signaling. We examined the molecular structure, biophysical properties, and functional roles of voltage-gated Na^+^ (Na_V_), Ca^2+^ (Ca_V_), and K^+^ (K_V_) channels in vascular endothelial cells. Particular attention was given to studies investigating VGIC activity in native endothelium and to emerging mechanisms regulating their activation. Endothelial cells express multiple VGIC subtypes at low densities, which are insufficient to generate action potentials but can modulate membrane potential (V_M_) and Ca^2+^-dependent signaling. The dynamic regulation of the endothelial V_M_, through the interplay between hyperpolarizing and depolarizing conductances, emerges as a key determinant of VGIC availability and activation. VGICs contribute to essential endothelial functions, including angiogenesis, vasomotor responses, blood–brain barrier permeability, and inflammation. Dysregulated VGIC expression and/or activity may be implicated in several pathological conditions, such as atherosclerosis, calcific aortic stenosis, and tumor vascularization. VGICs represent an unexpected but functionally relevant component of endothelial signaling. Elucidating their role in native vascular beds and disease contexts may uncover novel mechanisms of endothelial regulation and identify new therapeutic targets in cardiovascular and cancer biology.

## 1. Introduction

Voltage-gated ion channels (VGICs), including those selective for sodium (Na^+^), calcium (Ca^2+^), and potassium (K^+^), are critical for initiating and propagating electrical signals, i.e., action potentials, induced by membrane depolarization in excitable cells, such as neurons, muscle fibers, and neuroendocrine cells [1,2]. Moreover, Ca^2+^ entering through voltage-gated Ca^2+^ channels (VGCCs) also functions as a second messenger, thereby triggering multiple physiological processes, including contraction, secretion, and gene expression [3]. Nevertheless, it is now clear that VGICs are also expressed at low densities in a broad variety of non-excitable cells, such as immune cells, astrocytes, chondrocytes, fibroblasts, red blood cells, mesenchymal stem cells, and cancer cells [4,5]. The densities of voltage-gated Na^+^ and Ca^2+^ channels are not sufficient to generate an action potential in response to depolarizing stimuli, but these channels can fine-tune cellular activity through local electrical (e.g., depolarization) or chemical (e.g., Ca^2+^) signals [4,5]. The documented expression of VGICs in non-excitable cells indicates that they regulate a broader spectrum of physiological functions, including phagocytosis, cytokine release, motility, immune responses, and bone formation [4,5]. In accord, emerging evidence suggests that VGICs can also contribute to multiple pathologies that are not canonically regarded as channelopathies [4,5,6], such as inflammatory disorders, several types of cancer, atherosclerosis, calcific aortic stenosis, and osteoarthritis.

The endothelium forms the innermost monolayer of all blood and lymphatic vessels and therefore constitutes the largest organ system in the human body, with a cumulative surface area of approximately 4000 m^2^. The estimated number of vascular endothelial cells ranges from 1 to 6 × 10^13^ [7,8,9]. Vascular endothelial cells serve as a dynamic signal transduction platform that integrates a myriad of chemical and physical cues that are generated both by parenchymal cells and by the bloodstream through numerous depolarizing and hyperpolarizing ion channels [10,11,12,13,14,15,16,17,18,19,20,21,22,23,24]. Vascular endothelial cells are typically considered electrically unexcitable, as they have never been observed to generate action potentials [25,26]. Nevertheless, endothelial cells from several vascular districts express multiple types of VGICs that regulate critical vascular functions, such as vasorelaxation, angiogenesis, and inflammation [25,26]. Herein, we first summarize the molecular structure and biophysical properties of the classical VGICs. We then describe the expression pattern and physiological roles of voltage-gated Na^+^, Ca^2+^, and K^+^ channels in vascular endothelial cells. Finally, we discuss the potential mechanisms underlying endothelial VGIC regulation. If confirmed in physiopathologically relevant animal and human models, the regulation of cardiovascular homeostasis by endothelial VGICs could open a new avenue for endothelial biomedicine [27,28,29,30,31,32,33,34].

## 2. Voltage-Gated Ion Channels: Molecular Structure and Biophysical Properties

The VGIC superfamily includes the voltage-gated Na^+^ (Na_V_), voltage-gated Ca^2+^ (Ca_V_), and voltage-gated K^+^ (K_V_) channels, which open in response to membrane depolarization. VGICs are among the most extensively exploited drug targets for cardiovascular, neurological, and immunological disorders; however, their broad tissue distribution has limited their therapeutic use compared to G protein-coupled receptors and intracellular kinases [35]. The VGIC superfamily comprises at least 143 genes, many of which encode voltage-independent channels, such as Transient Receptor Potential (TRP) channels, inwardly rectifying potassium (K_IR_) channels, two-pore domain potassium (K_2_P) channels, and ryanodine receptors (RyRs). These voltage-independent channels are not considered in the present review.

### 2.1. Voltage-Dependent Na^+^ Channels (Na_v_)

**Figure 1:**
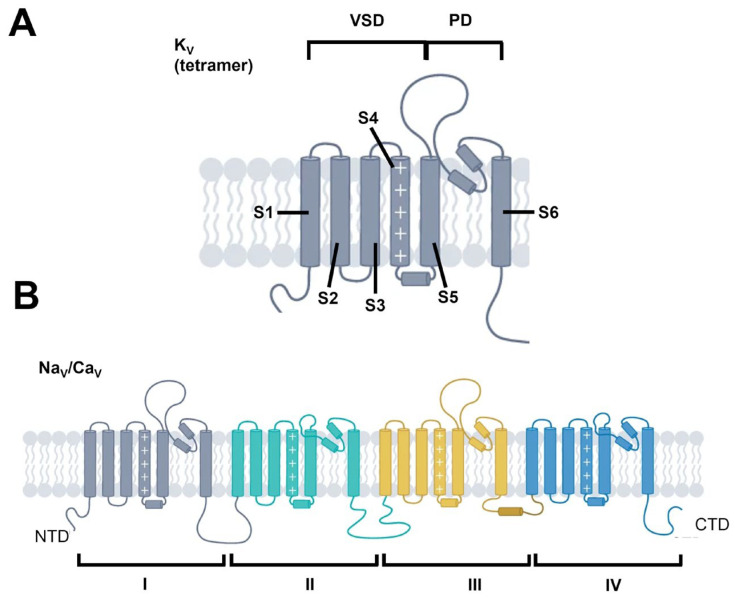
Topological structure of Na_V_/Ca_V_ channels. (**A**), the core subunit of a K_V_ channel consists of a tetramer formed by four protomers, each comprising six α-helices (from S1 to S6). Within each protomer, segments S1–S4 constitute the voltage-sensing domain (VSD), whereas segments S5 and S6, together with the intervening pore loop, contribute to the pore domain (PD). (**B**), the core subunit of Na_V_ and Ca_V_ channels is composed of a single polypeptide chain comprising four homologous repeats. Each repeat consists of six transmembrane α-helical segments (S1–S6). Within each repeat, segments S1–S4 constitute the VSD, whereas segments S5 and S6, together with the intervening pore loop, contribute to the PD. The four pore-forming regions collectively surround and define the ion-conducting pore. This and all the other figures in the manuscript were generated by using Powerpoint and Bioicons (https://bioicons.com/).

In humans, nine pore-forming Na_v_ channel α subunits have been identified (Na_V_1.1–Na_V_1.9), which share a high degree of amino acid homology, indicative of a conserved domain and transmembrane architecture. Each pore-forming α subunit is composed of four homologous domains (DI–DIV), each containing six transmembrane segments (S1–S6) (Figure 1) [36,37]. These domains exhibit a similar structural organization and comprise a voltage-sensing domain (VSD; S1–S4), characterized by positively charged residues within the S4 helix, and a pore-forming region (S5–S6) that confers Na^+^ selectivity and undergoes partial opening following voltage-sensor activation in domains DI–DIII in response to membrane depolarization (Figure 1) [36]. In heterologous expression systems, expression of the Na_V_ α subunit alone is generally sufficient to generate Na^+^ currents (*I_Na_*) in most eukaryotic cells [36]. In vivo, however, Na_v_ channels function as part of a larger multiprotein, membrane-embedded signaling complex [38], with auxiliary beta subunits (β1–β4) playing a central role. These subunits consist of a large N-terminal extracellular domain, a single transmembrane segment, and a cytosolic C-terminal domain [39] and modulate channel expression and gating properties [36]. Na_V_ α subunits can be classified based on their tissue-specific expression patterns. Na_v_1.1–Na_V_1.3 and Na_V_1.6 are predominantly expressed in the central nervous system, whereas Na_V_1.4 and Na_V_1.5 are primarily associated with skeletal muscle and cardiac tissue, respectively. Na_V_1.7–Na_V_1.9 are mainly localized in dorsal root ganglion neurons. NaX, also referred to as Na_V_2.1 and encoded by the *SCN7A* gene, acts as a TTX-sensitive, non-selective cation channel [36,37]. Na_V_ channels can also be categorized based on their sensitivity to the neurotoxin tetrodotoxin (TTX): Na_V_1.1–Na_V_1.4, Na_V_1.6–Na_V_1.7 are TTX-sensitive, as they are inhibited by nanomolar concentrations of TTX, while Na_V_1.5, Na_V_1.8, and Na_V_1.9 are TTX-resistant, as they are blocked only when TTX reaches the micromolar range [1].

**Figure 2:**
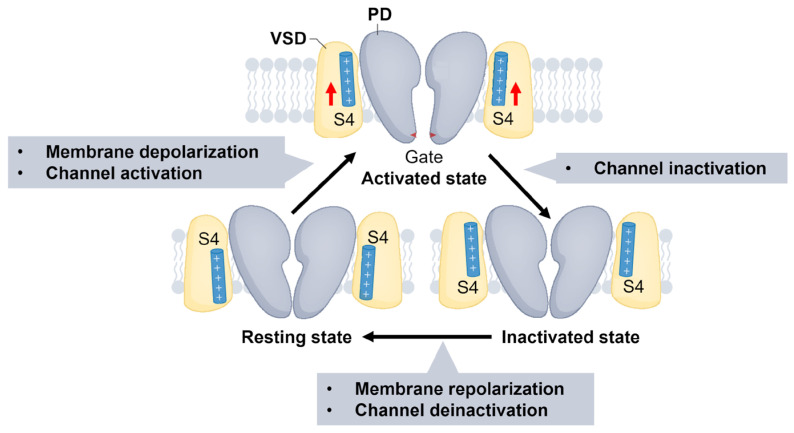
Voltage-gated ion channels are modulated by membrane depolarization. The resting state presents the voltage-sensing domains (VSDs) in the “down” conformation and the activation gate in a closed state (**bottom left**). Upon membrane depolarization, the gating charges within the S4 segment move toward the extracellular side, resulting in the transition of the VSDs to the “up” (activated) conformation and opening of the activation gate (**top**). The movement of helix S4 is shown here as a representative example of VSD activation.

In general, Na_V_ channels are rapidly activating and inactivating channels, typically closing within 1–2 ms (Figure 2) [37]. However, under specific conditions, incomplete fast inactivation may give rise to persistent *I_Na_*. The Na_V_1.9 subtype is well known for generating low-threshold persistent *I_Na_* in sensory neurons. In addition, Na_V_1.4 and Na_V_1.6 have been reported to produce persistent *I_Na_* in skeletal muscle fibers and Purkinje neurons, respectively [40]. In certain neuronal populations, Na_v_ channels can reopen during membrane repolarization as they recover from fast inactivation, producing a transient inward *I_Na_* known as the resurgent current. This current is thought to arise from the relief of open-channel block mediated by auxiliary β subunits [40]. Moreover, recent findings have shown that gain-of-function mutations in Na_V_1.7 enhance resurgent currents in dorsal root ganglion neurons [41].

### 2.2. Voltage-Dependent Ca^2+^ Channels (Ca_V_)

In humans, ten pore-forming α1 subunits have been identified, and the resulting Ca_V_ channels can be grouped into three subfamilies: Ca_V_1 channels (Ca_V_1.1–Ca_V_1.4), also known as L-type channels because of their long-lasting and large currents; Ca_V_2 channels (Ca_V_2.1–Ca_V_2.3), which comprise P/Q (Purkinje)-type (Ca_V_2.1), N (non-L and neuronal)-type (Ca_V_2.2), and R-type (resistant to dihydropyridines and P/Q/N toxins) channels; and Ca_v_3 channels (Ca_V_3.1–Ca_V_3.3), also referred to as T (transient and tiny)-type channels [1,3]. Ca_V_1.1-Ca_V_1.4 α1 subunits are encoded by the *CACNA1S*, *CACNA1C*, *CACNA1D*, and *CACNA1F* genes; Ca_V_2.1–Ca_V_2.3 α1 subunits are encoded by the *CACNA1A*, *CACNA1B*, and *CACNA1E* genes; Ca_V_3.1–Ca_V_3.3 α1 subunits are encoded by the *CACNA1G*, *CACNA1H*, and *CACNA1I* genes [42]. The Ca_V_ channels consist of the pore-forming α1 subunit and the auxiliary α2δ, β, and γ subunits. The α1 subunit consists of four homologous domains (DI–DIV), each containing six transmembrane segments (S1–S6), with the S4 segments acting as voltage sensors and the S5–S6 regions forming the Ca^2+^-selective pore (Figure 1). The extracellular α2δ and intracellular β subunits modulate channel trafficking, gating kinetics, and voltage dependence of Ca_V_1 and Ca_V_2 channels, whereas Ca_V_3 channels do not require auxiliary subunits to function [3,42].

Distinct Ca_V_ α1 isoforms display specific biophysical and pharmacological properties, including differences in the kinetics and voltage dependence of channel activation (Figure 2), inactivation (Figure 2), and single-channel conductance. The Ca_V_1 and Ca_V_2 families require strong membrane depolarization (typically >−20 mV) and are therefore classified as high-voltage-activated (HVA) channels, whereas the Ca_V_3 family is considered low-voltage-activated (LVA), as these channels respond to relatively small changes in membrane potential (V_M_; typically > −55 mV) [3,42]. HVA channels generally activate at membrane potentials ranging from 0 to −40 mV, whereas LVA channels are active at more negative potentials, typically between −50 and −70 mV. Moreover, LVA channels exhibit faster inactivation kinetics, whereas HVA channels are characterized by slower or delayed inactivation. A key feature of LVA channels is that the overlap between activation and inactivation curves generates a voltage window near the resting V_M_, within which the channels are transiently active yet partially inactivated. However, at the resting V_M_, a substantial fraction of LVA channels remain in an inactivated state and are therefore unavailable for opening; consequently, a brief membrane hyperpolarization can promote recovery from inactivation, thereby increasing channel availability. Upon subsequent membrane depolarization, a larger pool of channels can be recruited, resulting in a marked increase in T-type current amplitude [3,42,43]. Ca_V_1 channels are sensitive to Ca^2+^ antagonist drugs, such as dihydropyridines, benzothiazepines, and phenylalkylamines, which preferentially target Ca_V_1.2 channels and are widely used in the treatment of cardiovascular disorders [44]. Ca_V_2 channels are insensitive to inorganic L-type Ca^2+^ channel blockers but are inhibited by peptide toxins isolated from predatory animals, such as spiders and marine mollusks: N-type currents are blocked by the cone snail peptide ω-conotoxin GVIA and related peptide toxins; P-type currents are sensitive to the spider toxin ω-agatoxin IVA, whereas Q-type currents are inhibited by SNX-482, a peptide isolated from the venom of the African tarantula (*Hysterocrates gigas*). Ca_V_3 channels are potently inhibited by divalent metal ions, including nickel and zinc, as well as by trivalent metal ions, such as lanthanides, and by peptide toxins, such as Protoxins I and II, peptides isolated from the *Thrixopelma pruriens* tarantula, and PsPTx3, which is isolated from *Theraphosidae* tarantulas [3,44,45].

### 2.3. Voltage-Dependent K^+^ Channels (K_V_)

The human genome contains at least 70 K^+^ channel genes, 40 of which encode the voltage-dependent K_V_ pore-forming α subunits. K_V_ channels are subdivided into 12 subfamilies, designated as K_V_1 to K_V_12, based on sequence homology and functional properties: *KCNA* (K_V_1 family), *KCNB* (K_V_2), *KCNC* (K_V_3), *KCND* (K_V_4), *KCNF* (K_V_5), *KCNG* (K_V_6), *KCNQ* (K_V_7), *KCNV* (K_V_8), and *KCNS* (K_V_9), and *KCNH* [K_V_10, K_V_11, and K_V_12, including ether-a-go-go-related gene (EAG) and human ether-a-go-go-related gene (ERG)] [46]. Although K_V_ channels can form homotetrameric complexes, they are also able to assemble as heterotetramers through the combination of different α subunits. These heteromeric channel assemblies may display distinct voltage- and time-dependent gating properties compared with their homotetrameric counterparts [1,46]. However, K_V_5, K_V_6, K_V_8, and K_V_9 subunits are known as electrically silent because they do not form functional, ion-conducting channels on their own when expressed in heterologous systems, but instead combine with K_V_2 subfamily subunits, thereby attenuating K_V_2-mediated currents [1,46]. Each K_V_ α subunit consists of six transmembrane segments (S1–S6) (Figure 1), with the S4 segment acting as the principal voltage sensor and the S5–S6 region forming the ion-conducting pore (Figure 1). K_V_β subunits, KChIPs, DPP-like proteins, KCNE family members, such as minK, MiRPs, KCNE1-like proteins, and γ subunits (including LRRC26) constitute the major classes of K_V_ auxiliary subunits. These auxiliary subunits regulate the assembly, trafficking, and gating behavior of α subunits in a manner analogous to that described for Na_V_ and Ca_V_ channels [1,46]. Electrophysiological recordings have shown that voltage-gated K^+^ currents (*I_K_*) can be classified into delayed rectifier (K_DR_) and A-type (K_A_) currents, which activate after a short delay following membrane depolarization to a threshold of-35 mV and -65 mV, respectively [47]. K_A_ and K_DR_ currents have been defined primarily based on the kinetics and extent of channel inactivation during a depolarizing step. Thus, K_A_ currents, mainly mediated by K_V_1.4, K_V_3.3, K_V_3.4, K_V_4.1, K_V_4.2, and K_V_4.3 subunits, display rapid inactivation, whereas K_DR_ currents, which are carried by K_V_1.1, K_V_1.2, K_V_1.3, K_V_1.5, K_V_1.6, K_V_2.1, K_V_2.2, K_V_3.1, K_V_3.2, K_V_7.1, K_V_7.2, K_V_7.3, K_V_7.4, and K_V_7.5 family members, exhibit little or no appreciable inactivation over the course of a 100–200 ms depolarization. The steady-state inactivation properties of these currents are also distinct, such that K_A_ currents are largely inactivated at membrane potentials depolarized to approximately −50 mV, whereas K_DR_ currents do not show pronounced steady-state inactivation [1,46]. The subunit composition determines the pharmacology of K_V_ channels. K_A_ currents are inhibited by 4-aminopyridine (4-AP), whereas they are insensitive to tetraethylammonium (TEA). In contrast, K_DR_ currents are blocked by moderate concentrations of TEA (EC_50_ ranging from 0.3 to 560 mM in K_V_1.1 and K_V_1.2 channels, respectively) [46]. The detailed pharmacological profile of K_V_ channels can be found in [39,48].

## 3. Voltage-Gated Ion Channels in Vascular Endothelial Cells

Vascular endothelial cells are generally considered non-excitable cells, and it is therefore challenging to reconcile the expression of VGICs with the slow and often modest fluctuations in membrane potential observed in these cells. Nevertheless, multiple studies have convincingly demonstrated the presence of VGICs in both cultured and freshly isolated endothelial cells [25,49]. The expression pattern and functional role of the voltage-gated Na^+^, Ca^2+^, and K^+^ channels described in vascular endothelial cells are illustrated in Table 1, Table 2 and Table 3.

### 3.1. Endothelial Na_V_ Channels

Na_V_ subunits and currents have been reported in endothelial cells isolated from multiple vascular districts (Table 1). The first electrophysiological evidence of an endothelial *I_Na_* was provided by Gordienko and Tsukahara, who recorded TTX-resistant *I_Na_* in cultured endothelial cells derived from the rat interlobar artery (RIAE cells) [50]. Consistently, the dose–response relationship showed that the dissociation constant (K_d_) for TTX blockade was 1.4 μM [50], whereas the K_d_ of TTX-sensitive *I_Na_* is around 10 nM [51]. The half-maximal voltage (V_0.5_) for Na_V_ channel inactivation was −81.8 mV, whereas the resting V_M_ was approximately −38 mV [50]. These findings suggest that *I_Na_* in RIAE cells can only be activated following strong membrane hyperpolarization. Their pharmacological profile further indicates that these *I_Na_* are mediated by a TTX-insensitive Na_V_ subunit, such as the cardiac Na_V_1.5 isoform [1]. *I_Na_* were then recorded in rat cardiac microvascular endothelial cells (CMECs). Unlike those recorded in RIAE cells, these currents were TTX-sensitive, as the K_d_ for Na_v_ channel block was 5 nM [52]. Moreover, the V_0.5_ for inactivation was −45 mV [52], whereas the resting V_M_ of rat CMECs ranges between −40 mV [52] and −20 mV [53]. Therefore, the percentage of Na_V_ channels that are not inactivated and could be recruited by membrane depolarization varies from 30% to 15% [52]. Interestingly, *I_Na_* were recorded after the first day of CMEC isolation, which suggests that Na_V_ channels are present in cardiac microvessels in vivo [52]. While providing the first evidence that *I_Na_* were detectable in vascular endothelial cells, these studies did not report Na_V_ channel expression and only speculated about their physiological role. A TTX-resistant *I_Na_* was also recorded in human saphenous vein endothelial cells (HSVECs) [54]. Accordingly, the IC_50_ for TTX was 4.7 μM. The V_0.5_ for inactivation was ≈−75 mV, whereas the resting V_M_ of HSVECs was found to be ≈−28 mV. Notably, RT-PCR and immunoblotting detected the expression of the Na_V_1.5 subunit in both cultured HSVECs and intact saphenous venous endothelium [54].

**Figure 3:**
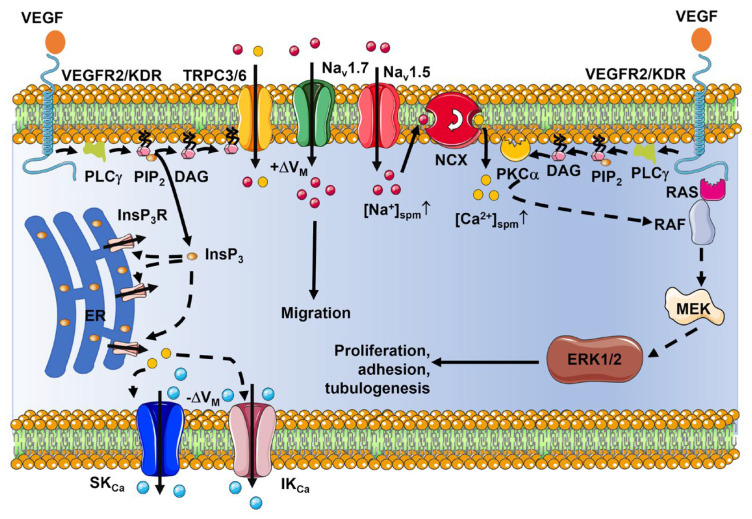
Endothelial cells Na_V_1.5 and Na_V_1.7 channels support VEGF-induced angiogenic activity. VEGF binds to the tyrosine kinase receptor VEGFR2/KDR, which stimulates phospholipase Cγ (PLCγ). The mechanism coupling PLCγ to membrane depolarization and Na_v_ channel activation remains poorly defined. Based on the current evidence, PLCγ-dependent hydrolysis of phosphatidylinositol 4,5-bisphosphate (PIP_2_) into diacylglycerol (DAG) and inositol-1,4,5-trisphosphate (InsP_3_). The latter activates InsP_3_ receptors (InsP_3_Rs), thereby activating small- and/or intermediate-conductance Ca^2+^-activated K^+^ channels (SK_Ca_ and IK_Ca_, respectively) and inducing endothelial hyperpolarization (−ΔV_M_). Endothelial hyperpolarization, in turn, recovers *I_Na_* from inactivation. This enables Na_V_1.5 and Na_V_1.7 to be activated by the following depolarization, which is mediated by the DAG-sensitive non-selective cation channels Transient Receptor Potential Canonical 3 (TRPC3) and TRPC6 [60,61]. Na_V_1.7 stimulates endothelial migration through a yet-to-be-identified mechanism. Extracellular Na^+^ entry through Na_V_1.5 channels causes an elevation in the sub-plasma membrane Na^+^ concentration ([Na^+^]_spm_), which shifts the Na^+^/Ca^2+^ exchanger (NCX) into the Ca^2+^ entry mode (3 Na^+^ out: 1 Ca^2+^ in), thereby resulting in a sub-plasma membrane Ca^2+^ microdomain ([Ca^2+^]_spm_). Intracellular Ca^2+^ recruits protein kinase Cα (PKCα) to the plasma membrane, where it is activated by DAG. PKCα, in turn, phosphorylates B-RAF to engage the extracellular signal-regulated kinase 1/2 (ERK1/2) pathway. Red circles: sodium ions. Yellow circles: calcium ions. Blue circles: potassium ions.

Subsequent investigations detected Na_V_1.2, Na_V_1.6, and Na_V_1.9 channels in the intact endothelium of mouse cremaster arterioles [55], Na_V_1.7 in endothelial cells from human cutaneous arterioles [56], Na_V_1.5, Na_V_1.7, Na_V_ channel β1, and Na_V_ channel β3 in human umbilical vein endothelial cells (HUVECs) (Table 1) [57]. Whole-cell patch-clamp recordings showed that HUVECs express both TTX-resistant (IC_50_ > 200 nM) and TTX-sensitive (IC_50_ = 1–10 nM) *I_Na_*, which could be mediated by Na_V_1.5 and Na_V_1.7 channels, respectively [58]. Functional analysis showed that vascular endothelial growth factor (VEGF) required Na_V_1.7 activity to promote migration and Na_V_1.5 activity to induce substrate adhesion, proliferation, and tubulogenesis (Figure 3) [58]. Na_V_1.5 was then found to enhance the activation of the extracellular signal-regulated kinase (ERK1/2) pathway (Figure 1) [58], which plays a crucial role in VEGF-induced angiogenesis [22]. VEGF-dependent ERK1/2 activation required the Ca^2+^-dependent recruitment of protein kinase Cα (PKCα), which in turn activates the ERK1/2 pathway through B-RAF (Figure 3) [58]. This report did not measure the V_0.5_ for *I_Na_* inactivation, whereas the resting V_M_ of HUVECs is approximately −30 mV [50]. However, voltage-sensitive dye imaging demonstrated that VEGF causes transient hyperpolarization [58], which is mediated by large-conductance Ca^2+^-activated K^+^ channels (BK_Ca_) [59] and is likely to recover a fraction of Na_v_ channels from inactivation. Consistently, this hyperpolarization was followed by a sustained depolarization [58]. The late VEGF-induced depolarization is predicted to bring V_M_ to the activation threshold and thereby activate Na_V_1.5 channels in HUVECs. This depolarization caused the reversal of the Na^+^/Ca^2+^ exchanger (NCX) into the Ca^2+^ entry mode, which generated the submembrane Ca^2+^ microdomain responsible for VEGF-induced PKCα recruitment (Figure 3) [58]. These findings indicate that Na_V_1.5 and Na_V_1.7 support the endothelial pro-angiogenic signaling machinery. Future studies should assess whether Na_V_ channels are also expressed in the intact human umbilical vein and whether *I_Na_* is involved in VEGF-induced angiogenesis in other endothelial cell types.

Additionally, endothelial Na_V_ channels—Na_V_1.2, Na_V_1.6, and Na_V_1.9—have been suggested to control the vasomotor tone in the microcirculation (Table 1). Focal electrical stimulation of mouse cremaster arterioles evoked a conducted vasodilation on both sides of the stimulation point that spread without decay along the entire microvessel [55]. The non-decremental conducted vasodilation required an intact endothelium, was suppressed by blocking Na_V_ channels with TTX or bupivacaine and was mimicked by the Na_V_ channel agonist veratridine [55]. Furthermore, the vasomotor response was converted into vasoconstriction by loading endothelial cells with the membrane-permeable Ca^2+^ buffer BAPTA, whereas it was strongly attenuated by the pharmacological or genetic blockade of eNOS activity [55]. Finally, the conducted vasodilation was impaired in transgenic mouse models lacking Ca_V_3.2 channels. These findings suggest that local endothelial depolarization may activate endothelial Na_v_ channels to trigger a regenerative vasodilatory signal, which requires voltage-gated Ca^2+^ entry and eNOS activation [55]. This elegant model has been the subject of some controversy for the following reasons: (1) the biophysical properties of Na_V_1.2, Na_V_1.6, and Na_V_1.9 channels have not been measured; (2) the V_0.5_ for inactivation of *I_Na_*, which determines the fraction of Na_V_ channels that can be activated under resting conditions, is unknown; and (3) the endothelial expression of Ca_V_3.2 channels in mouse cremaster arterioles could not be confirmed by immunocytochemistry.

**Figure 4:**
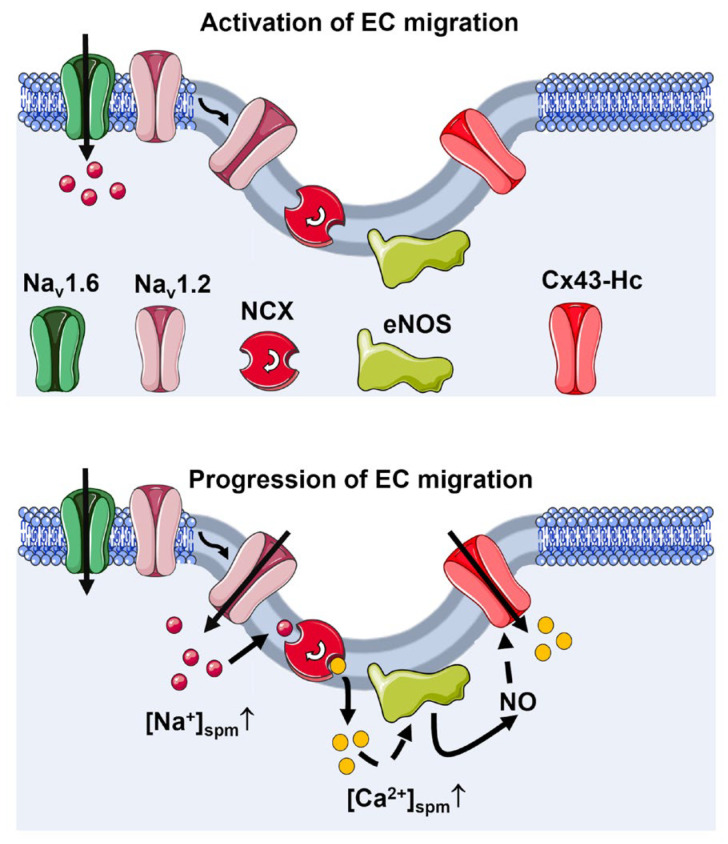
Na_V_1.6 and Na_V_1.2 regulate endothelial cell migration. (**Top panel**): scratching the endothelial cell monolayer in the wound-healing assay activates Na_V_1.6, thereby promoting Na_V_1.2 translocation within the adjacent caveolae. (**Bottom panel**): within the caveolae, Na_V_1.2 is positioned in close proximity with the Na^+^/Ca^2+^ exchanger (NCX). Na_V_1.2-mediated Na^+^ entry causes an elevation in the sub-plasma membrane Na^+^ concentration ([Na^+^]_spm_), which shifts the Na^+^/Ca^2+^ exchanger (NCX) into the Ca^2+^ entry mode (3 Na^+^ out: 1 Ca^2+^ in), thereby resulting in a sub-plasma membrane Ca^2+^ microdomain ([Ca^2+^]_spm_). This Ca^2+^ microdomain stimulates the endothelial nitric oxide (NO) synthase (eNOS) to release NO, which causes the opening of Cx43 hemichannels (Cx43-Hcs) by NO-mediated S-nitrosylation. Ca^2+^ entry through Cx43-Hcs amplifies the Ca^2+^ signal driving endothelial cell migration. Red circles: sodium ions. Yellow circles: calcium ions.

Na_V_1.2 and Na_V_1.6 channels may also drive migration in rat mesenteric endothelial cells (Table 1). Mechanical disruption of the endothelial monolayer initiates cell migration by activating Na_V_1.6 channels, which subsequently promote the recruitment of Na_V_1.2 channels to caveolae (Figure 4) [62]. Within caveolae, Na_V_1.2 channels interact with caveolin-1, thereby positioning them in close proximity to the NCX [62]. As a result, Na_V_1.6-dependent activation of Na_V_1.2 channels increases local Na^+^ concentration within the caveolar microdomain, consequently stimulating Ca^2+^ entry via the reverse mode of NCX (Figure 4) [62]. NCX-mediated Ca^2+^ entry recruits eNOS and promotes the opening of Cx43 hemichannels through NO-dependent S-nitrosylation (Figure 4) [63]. Since Cx43 hemichannels are permeable to Ca^2+^ [64,65], their activation further amplifies the intracellular Ca^2+^ signal required for endothelial cell migration [62]. This investigation did not evaluate the biophysical properties of Na_V_1.2 and Na_V_1.6 channels. However, endothelial wounding promotes cation entry through uncoupled connexins, thereby potentially leading to endothelial depolarization [66].

Finally, a recent transcriptomic analysis revealed that Na_V_1.5, Na_V_1.6, and Na_V_1.7, together with the ancillary β1 subunit, are expressed in human brain microvascular endothelial cells (BMECs) (Table 1) [67]. It remains to be elucidated whether these transcripts encode functional Na_V_ channels and their signaling role. Emerging evidence suggests that endothelial ion signaling plays a crucial role in the regulation of cerebral blood flow (CBF) [16,68] and synaptic activity [69,70]. Notably, a recent investigation showed that endothelial depolarization may contribute to promoting CBF recovery to the baseline, thereby fine-tuning the hemodynamic response to neuronal activity [71]. Future work should assess whether endothelial Na_V_ channels contribute to the upstream propagation of this depolarizing signal from the activated brain capillaries.

In conclusion, available evidence supports the notion that Na_V_ channels are expressed in both cultured endothelial cells and the intact endothelium of naïve vessels. Two main questions need to be addressed. First, are *I_Na_* involved in endothelial functions other than angiogenesis and the control of vascular tone? Second, since the resting V_M_ of vascular endothelial cells is rather depolarized as compared with that of excitable cells, what physiological mechanisms enable Na_V_ channel recruitment when they are in an inactive state? This issue, which was initially addressed in [58], will be discussed in Section 4.

**Table 1:** Na_V_ channels and currents in vascular endothelial cells.

Na_V_ Subunit	EC Type	Detection Method	Function and Mode of Evidence	Biophysical Properties (V_t_, V_0.5act_, V_0.5inact_)	Confidence	Ref.
Na_V_1.2	Mouse CAECs	IF (in situ)	Arteriolar tone–Pharmacological	Undefined	Low	[55]
Na_V_1.2	Rat MECs	IF (cultured)	Migration–Pharmacological	Undefined	Low	[62]
Na_V_1.5	HSVECs	RT-PCR (cultured), IF In situ)	Undefined–Patch-clamp	V_0.5act_ ≈ −30 mV, V_0.5inact_ ≈ −75 mV	High	[54]
Na_V_1.5	HUVECs	RT-PCR (cultured)	Angiogenesis–Patch-clamp and pharmacology	V_T_ ≈ −40 mV	High	[58]
Na_V_1.5	Human BMECs	RT-PCR (cultured)	Undefined	Undefined	Low	[67]
Na_V_1.6	Mouse CAECs	IF (in situ)	Arteriolar tone–Pharmacology	Undefined	Low	[55]
Na_V_1.6	Rat MECs	IF (cultured)	Migration–Pharmacology	Undefined	Low	[62]
Na_V_1.6	Human BMECs	RT-PCR (in vitro)	Undefined	Undefined	Low	[67]
Na_V_1.7	HUVECs	RT-PCR (cultured)	Angiogenesis–Patch-clamp and angiogenesis	V_T_ ≈ −40 mV	High	[58]
Na_V_1.7	Human CuAECs	IF (in situ)	Undefined	Undefined	Low	[56]
Na_V_1.7	Human BMECs	RT-PCR (cultured)	Undefined	Undefined	Low	[67]
Na_V_1.9	Mouse CAECs	IF (in situ)	Arteriolar tone–Pharmacology	Undefined	Low	[55]
Undefined	RIAECs	Electrophysiology (cultured)	Undefined–Patch clamp and pharmacology	V_0.5act_ = −37.5 mV, V_0.5inact_ = −81.8 mV	High	[50]
Undefined	Rat CMECs	Electrophysiology (cultured)	Undefined–Patch clamp and pharmacology	V_T_ = −20 mV, V_0.5inact_ = −45 mV	High	[52]

Abbreviations: BMECs: brain microvascular endothelial cells; CAECs: cremaster arteriole endothelial cells; CMECs: cardiac microvascular endothelial cells; EC: endothelial cell; HSVECs: human saphenous vein endothelial cells; IF: immunofluorescence; MECs: mesenteric endothelial cells; HUVECs: human umbilical vein endothelial cells; RIAECs: rat interlobar artery endothelial cells; V_0.5act_ = half-maximum voltage for activation; V_0.5inact_ = half-maximum voltage for inactivation; V_T_: voltage-activation threshold.

### 3.2. Endothelial Ca_V_ Channels

Ca_V_ subunits and voltage-dependent Ca^2+^ currents (*I_Ca_*) have been reported in endothelial cells isolated from capillaries but not from large vessels (Table 2). *I_Ca_* have been recorded in microvascular endothelial cells from bovine adrenal glands (BAMCECs) [72], rat and pig brains [73,74], and rat and mouse lungs [49]. The following features indicate that the *I_Ca_* measured in BAMCECs are mediated by L- and T-type channels resembling those described in endocrine secretory cells: distinct kinetic and gating properties, characteristic current–voltage (I/V) relationships, pharmacological sensitivity to nifedipine, BAY K 8644, and amiloride, as well as differential permeability to Ca^2+^ and Ba^2+^ [72,75]. Moreover, T-type currents exhibited a single-channel conductance of ≈10 pS, whereas L-type currents displayed a single-channel conductance of ≈16 pS [75]. The steady-state activation (V_0.5_ ≈ −28 mV) and inactivation (V_0.5_ ≈ −33 mV) curves of T-type currents showed a partial overlap between −50 mV and 0 mV, thereby producing a sustained window current centered at −30 mV [72]. The resting V_M_ of BAMCECs was ≈−50 mV [75]; thus, a small depolarization is predicted to evoke a robust influx of Ca^2+^ through T-type Ca_V_ channels [72]. Ca^2+^ imaging recordings confirmed that BAMCEC depolarization induced by elevated extracellular K^+^ caused a transient increase in intracellular Ca^2+^ concentration ([Ca^2+^]_i_) that was sensitive to cadmium, a potent blocker of T-type Ca^2+^ channels [75]. In addition to T-type and L-type *I_Ca_*, BAMCECs also express a distinct HVA Ca^2+^ channel, referred to as SB, which displays an activation threshold of −10 mV, exhibits a single-channel conductance of 2.8 pS in 100 mmol/L Ba^2+^, and is activated by BAY K 8644 while remaining insensitive to nicardipine [76]. Subsequent work showed that acetylcholine gates nicotinic receptors, thereby depolarizing the BAMCECs and evoking voltage-dependent Ca^2+^ entry through both LVA and HVA channels [77]. It should, however, be noted that the molecular expression of Ca_V_ subunits in BAMCECs remains to be defined by RT-PCR and/or immunofluorescence analysis.

Rat pulmonary microvascular endothelial cells (PMVECs) exhibit a T-type *I_Ca_*, which is transiently activated at V_M_ of approximately −60 mV and reaches a peak at −10 mV [78]. The estimated V_0.5_ values for activation and inactivation were −28.4 mV and −51.4 mV, respectively [78]. The overlap between the steady-state activation and inactivation curves predicts the presence of a “window current” spanning the membrane potentials between −60 and −30 mV. Furthermore, PMVEC *I_Ca_* was sensitive to established T-type channel inhibitors, including mibefradil, flunarizine, pimozide, and the scorpion toxin kurtoxin [78]. RT-PCR analysis confirmed that rat PMVECs expressed the pore-forming Ca_V_3.1 subunit, as well as the α2δ2, β1 and β3, but not β2 and β4, auxiliary subunits [78,79]. Ca^2+^ imaging recordings further showed that Ca_V_3.1 T-type channels sustain Ca^2+^ entry elicited by agonists of G_q_ protein-coupled receptors (G_q_PCRs), such as thrombin [78]. It has therefore been proposed, although not demonstrated, that G_q_PCR stimulation induces transient hyperpolarization followed by depolarization, which are mediated by yet-to-be-identified Ca^2+^-activated K^+^ channels (K_Ca_) and store-operated channels (SOCs) (see Section 4.3), respectively [78,79,80].

**Figure 5:**
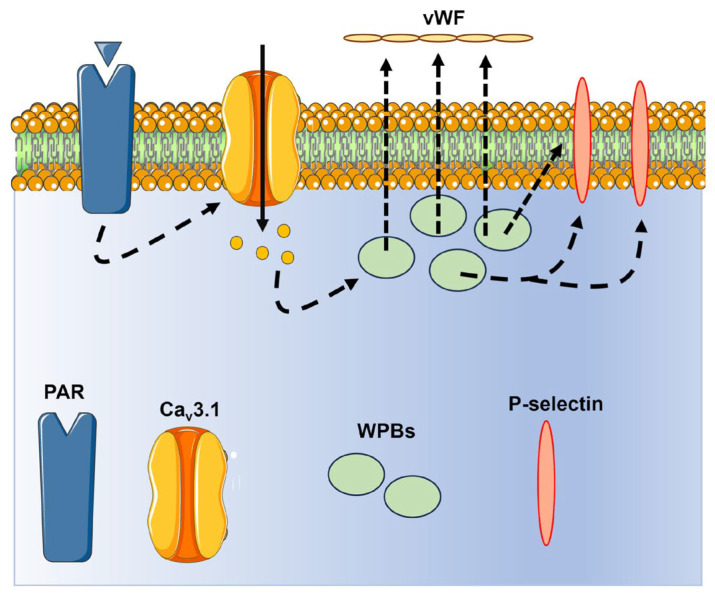
Endothelial Ca_V_3.1 channels control the exocytosis of von Willebrand factor and P-selectin from Weibel–Palade bodies. Thrombin binds to protease-activated receptor (PAR) to induce membrane depolarization, thereby activating Ca_V_3.1 channels and promoting Ca^2+^ entry. The resulting increase in [Ca^2+^]_i_ induces the rapid secretion of von Willebrand factor (vWF) and plasma-membrane expression of P-selectin from Weibel–Palade bodies (WPBs). Yellow circles: calcium ions.

The involvement of endothelial Ca_V_3.1 channels in the biphasic Ca^2+^ response to inflammatory mediators suggests that they play a crucial role in rat lung injury [79]. An increase in [Ca^2+^]_i_ triggers the exocytosis of Weibel–Palade bodies (WPBs), thereby delivering the adhesive protein von Willebrand factor (vWF) and the leukocyte adhesion molecule P-selectin to the cell surface, where they facilitate coagulation and inflammation [81,82]. Thrombin-induced vWF secretion and surface translocation of P-selectin in pulmonary capillary endothelium were inhibited by blocking the T-type *I_Ca_* with mibefradil or by genetically inhibiting Ca_v_3.1 expression (Figure 5) [83,84,85]. Accordingly, the pharmacological blockade of Ca_v_3.1 with mibefradil, flunarizine, and pimozide interfered with the retention of sickled erythrocytes in the inflamed lung microcirculation [78].

A subsequent investigation showed that the abrupt interruption of shear stress, as may occur with flow-cessation caused by vascular obstruction, embolism, or surgery, elicited membrane depolarization and Ca^2+^ entry supported by a T-type *I_Ca_* [86]. The Ca^2+^ response to flow-cessation can in turn stimulate NO production [87], which may function as a compensatory vasodilatory mechanism. Ca^2+^ entry through Piezo1 [88] and TRP Vanilloid 4 (TRPV4) channels [89] underlies the mechanosensitive recruitment of eNOS. It is therefore reasonable to hypothesize that Ca_V_3.1 is also physically associated with eNOS in rat lung microvascular endothelium, as reported in mice (see below). Endothelial T-type *I_Ca_* are also involved in pulmonary angiogenesis. SOCE and TRP channels constitute the major Ca^2+^ entry pathways in vascular endothelial cells during the angiogenic process [22,23]. Nevertheless, the inhibition of PMVEC T-type *I_Ca_* with NNC 55-0396 or the genetic blockade of Ca_V_3.1 expression using short hairpin RNA attenuated the capacity for network, as well as proliferation, cell–matrix adhesion, and migration [90]. Voltage-dependent Ca^2+^ entry through Ca_V_3.1 channels recruits two distinct Ca^2+^-dependent pro-angiogenic signaling pathways, namely phosphatidylinositol-3-kinase (PI3K)/Akt [90] and Ca^2+^/Calmodulin-dependent protein kinase IV (CaMKIV) [91]. The resting V_M_ of rat PMVECs is scattered between −70 mV and −5 mV [92], which is within the voltage range of the Ca_V_3.1 T-type *I_Ca_* (−60 mV to −30 mV) and can therefore result in basal Ca^2+^ entry that drives the angiogenic behavior [90].

A recent investigation exploited immunofluorescence to demonstrate that the Ca_V_3.1 subunit is also expressed in mouse pulmonary artery endothelial cells (PAECs), where it is colocalized with eNOS [93]. Furthermore, acetylcholine-induced Ca^2+^ signals and NO release in PAECs were significantly reduced by mibefradil and by the genetic knockdown of Ca_V_3.1 [93]. Accordingly, blocking the T-type *I_Ca_* impaired acetylcholine-induced vasorelaxation [93]. This investigation did not assess the biophysical properties of T-type currents. However, the resting V_M_ of mouse PAECs is ≈−43 mV [94]. Acetylcholine elicits a membrane hyperpolarization to ≈−70 mV [94], which can de-inactivate Ca_V_3.1, thereby causing a tail Ca^2+^ current responsible for eNOS activation [95]. This signaling pathway could be impaired by chronic hypoxia, which causes Ca_V_3.1 downregulation in freshly isolated rat PAECs [96]. Future work should evaluate the biophysical features of T-type *I_Ca_* in mouse PAECs, their pharmacological profile, and whether they are coupled with other vasorelaxing pathways, such as small- and intermediate-conductance Ca^2+^-activated K^+^ channels [12,21].

**Figure 6:**
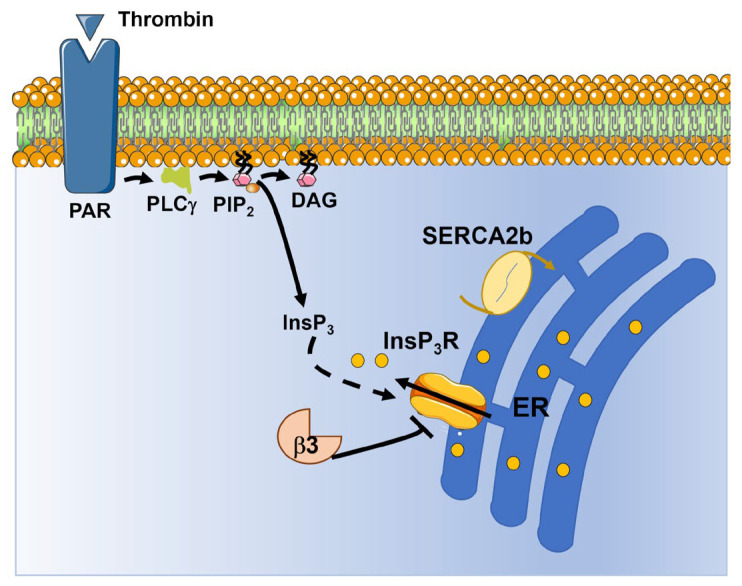
Endothelial Ca_V_β3 reduces ER Ca^2+^ release through InsP_3_Rs. The Ca_V_β3 subunit may interact with ER-embedded InsP_3_Rs in vascular endothelial cells, thereby reducing ER Ca^2+^ release following stimulation of protease-activated receptor (PAR) by thrombin. Yellow circles: calcium ions.

Finally, a T-type *I_Ca_* was recorded in rat [73,97], but not mouse, BMECs [98]. The Ca_V_β3 subunit is also expressed in both mouse [98] and human [67] BMECs. The Ca_V_β3 subunit may desensitize inositol-1,4,5-trisphosphate (InsP_3_) receptors (InsP_3_Rs), which constitute the major Ca^2+^ release channel in the endothelial ER [12], to low InsP_3_ concentrations (Figure 6) [99]. Consistently, while *I_Ca_* could not be recorded in mouse BMECs, the genetic knockdown of Ca_V_β3 enhanced thrombin-induced ER Ca^2+^ release through InsP_3_Rs, thereby facilitating cell contraction and increasing endothelial barrier permeability [98]. Enhanced ER Ca^2+^ mobilization was found to potentiate the Ca^2+^-dependent recruitment of myosin light chain (MLC) kinase [98], which phosphorylates MLC to regulate actin–myosin contractile activity [100]. In an experimental mouse model of autoimmune encephalomyelitis, Ca_V_β3^−/−^ animals exhibited a significantly earlier disease onset, accompanied by more severe clinical manifestations and a marked increase in T-cell infiltration compared with control mice [98].

Endothelial InsP_3_Rs also play a crucial role in the Ca^2+^-dependent recruitment of eNOS at the neurovascular unit, thereby modulating CBF [101,102,103] and synaptic plasticity [69,70]. Future studies could also assess whether neurovascular coupling and long-term potentiation are impaired by the selective deletion of the endothelial Ca_V_β3 subunit.

**Table 2:** Ca_V_ channels and currents in vascular endothelial cells.

Ca_V_ Subunit	EC Type	Detection Method	Function and Mode of Evidence	Biophysical Properties (V_t_, V_0.5act_, V_0.5inact_)	Confidence	Ref.
Ca_V_3.1	Rat PMVECs	RT-PCR (cultured), electrophysiology	Retention of sickled erythrocytes–Patch-clamp and pharmacology	T-type: V_0.5act_ ≈ −28.4 mV, V_0.5inact_ ≈ −51.4 mV	High	[78]
Ca_V_3.1	Rat PMVECs	WB (cultured), electrophysiology	vWF secretion–Patch clamp on wild type cells and cells transduced with a selective siRNA against Ca_v_3.1 and pharmacology	T-type: V_T_ = −60 mV	High	[83,85]
Ca_v_3.1	Rat PMVECs	RT-PCR (cultured)	Flow adaptation–Ca^2+^ imaging and pharmacology	Undefined	Moderate	[86]
Ca_V_3.1	Rat PMVECs	WB (cultured), electrophysiology	Angiogenesis–Patch clamp on wild type cells and cells transduced with a selective shRNA against Ca_v_3.1 and pharmacology	Undefined	High	[90,91]
Ca_V_3.1	Mouse PAECs	IF (in situ)	Vasorelaxation–Ca^2+^ imaging and functional assays conducted in wild type and Cav3.1^−/−^ mice and pharmacology	Undefined	High	[93]
Undefined	Rat BMECs	Electrophysiology	Undefined–Patch clamp	T-type: V_T_ = −50 mV, V_0.5inact_ ≈ −55 mV	Moderate	[73,97]
Undefined	BAMCECs	Electrophysiology	Undefined–Patch clamp and pharmacology	T-type: V_0.5act_ ≈ −28 mV, V_0.5inact_ ≈ −33 mV, SC γ ≈10 pS	High	[72,75]
Undefined	BAMCECs	Electrophysiology	Undefined–Patch clamp and pharmacology	L-type: V_0.5act_ ≈ −18 mV	High	[72]
Undefined	BAMCECs	Electrophysiology	Undefined–Patch clamp and pharmacology	SB-type: V_T_ = −10 mV, SC γ = 2.8 pS	High	[76]

Abbreviations: BAMCECs: bovine adrenal gland microvascular endothelial cells; EC: endothelial cell; PAECs: pulmonary artery endothelial cells; PMVECs: pulmonary microvascular endothelial cells; SC γ: single-channel conductance; shRNA: short hairpin RNA; siRNA: small interfering RNA; V_0.5act_: half-maximum voltage for activation; V_0.5inact_: half-maximum voltage for inactivation; WB: Western blotting.

### 3.3. Endothelial K_V_ Channels

K_V_ subunits and voltage-dependent K^+^ currents (*I_K_*) have been reported in endothelial cells from multiple vascular districts (Table 3). Electrophysiological recordings have demonstrated that vascular endothelial cells express both K_A_ and K_DR_ [104]. A fast-rising, rapidly inactivating outward *I_K_* elicited by depolarization with a threshold of −10 mV was first described in bovine aortic endothelial cells (BAECs) [105]. The transient outward current was inactivated at depolarized membrane potentials and was sensitive to 4-AP [105], which are features typical of K_A_ currents [104]. A depolarization-activated transient outward *I_K_* has also been identified in isolated capillary endothelial cells from the guinea pig heart [106]. This current exhibits an activation threshold positive to −20 mV and displays inactivation kinetics significantly slower than those of the A-type current observed in cultured BAECs [106]. 4-AP-sensitive K_A_-type currents have also been described in HUVECs [107] and rat BMECs [108], in which they are up-regulated by hypoxia through a CaMKII-dependent mechanism. However, the molecular expression of K_A_-type K_V_ subunits remains largely undefined (Table 3). Thus far, immunofluorescence has shown that K_V_1.4 is weakly expressed in mouse [109] and rat [110] brain arteriolar endothelial cells. Transcriptomic analysis revealed that human BMECs express the K_V_4.1 subunit and its auxiliary KChIP2 and KChIP3 subunits [67]. Future work should assess whether K_A_ currents and their underlying subunits are expressed in freshly isolated endothelial cells or in native endothelium in situ. The endothelial function of K_A_ currents also needs to be thoroughly investigated (Table 3). They contribute to setting the resting V_M_ in rat brain arteriolar endothelial cells [110]. Moreover, rapid K_A_ currents may function to restore the endothelial V_M_ following agonist-induced depolarization [104,106,111], resulting in V_M_ fluctuations in response to a prolonged depolarizing pulse [112,113,114,115]. Oscillations in the endothelial V_M_ may be synchronized with intracellular Ca^2+^ spikes by fine-tuning the electrochemical gradient for Ca^2+^ entry, thereby preventing Ca^2+^ overload [112,114]. Furthermore, endothelial V_M_ fluctuations may regulate vascular tone via endothelium-dependent hyperpolarization [12,112,116], capillary permeability [117], membrane stiffness [118], transcriptional activity [119], and angiogenesis [120]. Future work should assess whether and how the genetic deletion of K_A_-type currents affects both the endothelial V_M_ and endothelial functions.

Parallel studies have demonstrated that K_DR_-like currents are widely expressed in vascular endothelial cells (Table 3). A voltage-dependent *I_K_* that slowly activated upon membrane depolarization >−30 mV and showed little or no time-dependent inactivation during a sustained voltage step was measured in rat CMECs [52,121]. This K_DR_ current was sensitive to TEA and charybdotoxin; moreover, static stretch enhanced I_K_ amplitude and stimulated CMEC proliferation. This observation suggests that mechanical perturbation, which has long been known to stimulate cardiac angiogenesis [122], requires a fine-tuned regulation of the endothelial V_M_ by depolarizing Piezo1 channels [123] and hyperpolarizing K_DR_ currents [121]. K_DR_ currents have also been described in HUVECs [107], rat aortic endothelial cells [124], PAECs [125], and BMECs [110] (Table 3). Furthermore, several K_v_ subunits mediating K_DR_ currents have been detected in both cultured and native endothelial cells (Table 3). The role of K_DR_-like currents in vascular endothelial cells remains largely unknown. Their slow activation kinetics suggest that they could sustain endothelial repolarization following a depolarizing signal induced by either chemical or physical stimuli [112,113,114,115]. Furthermore, K_V_1.5 channels may be activated by mitochondrial ROS in human PAECs, thereby stimulating the secretion of NO, endothelin-1, and VEGF [126]. This finding suggests that the pharmacological blockade of K_V_1.5 channels represents a promising strategy to mitigate excessive ROS signaling in pulmonary artery hypertension [127]. Consistently, hydrogen peroxide (H_2_O_2_)-induced K_V_1.5 activation induced apoptosis in both HUVECs and in vivo rat carotid artery endothelial cells [128]. This signaling pathway was found to contribute to endothelial injury induced by oxidized low-density lipoprotein in atherosclerosis and by palmitate in type 2 diabetes mellitus. Notably, the specific K_V_1.5 inhibitor DPO-1 mitigated H_2_O_2_-induced endothelial apoptosis both in vitro and in vivo [128]. Moreover, ROS-dependent K_V_1.5 activation supports palmitate-induced endothelial injury in both HUVECs and rat thoracic aortic endothelium [129]. In agreement with these findings, it has long been known that the activation of K^+^ channels, including K_V_1.5 [127], stimulates early cell shrinkage and apoptosis [130]. K_DR_-like currents may also regulate BBB permeability. Retigabine, a specific K_V_7.3 agonist, reduced transendothelial electrical resistance in primary rat BMECs and counteracted the kainate-induced increase in permeability in an epileptic in vitro model of the BBB [131]. Finally, endothelial cells may express KCNE3, which is an auxiliary β-subunit that assembles with several K_V_ subunits, such as K_V_3.4 and K_V_7.1 [132]. KCNE3 has been put forward as novel marker of endothelial tip cells [133] and mouse embryonic capillary endothelial cells [134]. Lymph node metastatic melanoma induces the expression of the auxiliary KCNE4 β-subunit in tumor endothelium [135], which undergoes a dramatic remodeling of the ion signaling machinery [136], but its functional consequences are unknown.

**Table 3:** K_V_ channels and currents in vascular endothelial cells.

K_V_ Subunit	EC Type	Detection Method	Function and Mode of Evidence	Biophysical Properties (V_t_, V_0.5act_, V_0.5inact_)	Confidence	Ref.
K_V_1.3	Mouse brain arteriolar ECs	IF (in situ)	Arteriolar tone?	Undefined	Low	[109]
K_V_1.3	Rat brain arteriolar ECs	RT-PCR (cultured)	V_M_ modulation–Patch clamp and pharmacology	K_A_ (likely association with a β subunit); V_t_ = −40 mV	High	[110]
K_V_1.4	Mouse brain arteriolar ECs	IF (in situ)	Arteriolar tone?	Undefined	Low	[109]
K_V_1.4	Rat brain arteriolar ECs	RT-PCR (cultured)	V_M_ modulation?–Patch clamp and pharmacology	Undefined (weak expression)	High	[110]
K_V_1.5	RAECs	IF (in situ)	V_M_ modulation and apoptosis induction; Patch clamp and pharmacology, apoptotic assays carried out in cells treated with a selective siRNA	K_DR_; V_T_ = −20 mV	High	[124,129]
K_V_1.5	Rat PAECs	IF (in situ)	Undefined–Patch clamp	K_DR_; V_T_ ≈ −25 mV	High	[125]
K_V_1.5	Rat GECs	IF (in situ)	Undefined	Undefined	Low	[137]
K_V_1.5	Human PAECs	IF and WB (cultured)	ROS signaling–Functional assays conducted in cells treated with a specific siRNA	Undefined	Moderate	[126,128]
K_V_1.5	HUVECs	WB (cultured)	ROS signaling–Functional assays conducted in cells treated with a specific siRNA	Undefined	Moderate	[128,129]
K_V_1.6	Mouse brain arteriolar ECs	IF (in situ)	Arteriolar tone?	Undefined	Low	[109]
K_V_1.7	Rat GECs	IF (in situ)	Undefined	Undefined	Low	[137]
K_V_4.1	Human BMECs	RT-PCR (cultured)	Undefined	Undefined	Low	[67]
K_V_7.1	Rat BMECs	IF (cultured)	Undefined	Undefined	Low	[131]
K_V_7.4	Swine CAECs	IF (in situ)	Vasorelaxation–Functional assays and pharmacology	Undefined	Moderate	[138]
K_V_7.4	Rat BMECs	IF (cultured)	Reduces BBB permeability–Functional assays and pharmacology	Undefined	Moderate	[131]
K_V_7.5	Rat BMECs	IF (cultured)	Reduces BBB permeability–Functional assays and pharmacology	Undefined	Moderate	[131]
K_V_9.3	Human placenta arterial ECs	IF (in situ)	Undefined	Undefined	Low	[139]
Undefined	BAECs	Electrophysiology	Undefined–Patch clamp	K_A_; V_T_ = −10 mV;	High	[105]
Undefined	Guinea pig CMECs	Electrophysiology	Undefined–Patch clamp	K_A_; V_T_ ≈ −40 mV; V_0.5_ act = +7 mV; V_0.5_ inact = −50 mV	High	[106]
Undefined	Rat BMECs	Electrophysiology	Undefined–Patch clamp and pharmacology	K_A_: V_T_ = +5 mV; K_DR_: V_T_ = −10 mV	High	[108]
Undefined	HUVECs	Electrophysiology	Undefined–Patch clamp and pharmacology	K_A_: V_T_ = −20 mV; K_DR_ ≈ 0 mV	High	[107]

Abbreviations: CAECs: coronary artery endothelial cells; CMECs: coronary microvascular endothelial cells; EC: endothelial cells; GECs: glomerular endothelial cells; HUVECs: human umbilical vein endothelial cells; IF: immunofluorescence; PAECs: pulmonary artery endothelial cells; RAECs: rat aortic endothelial cells; ROS: reactive oxygen species; V_0.5act_: half-maximum voltage for activation; V_0.5inact_: half-maximum voltage for inactivation; V_T_: voltage-activation threshold; WB: Western blotting.

## 4. Physiological Stimulation of Voltage-Gated Ion Channels in Vascular Endothelial Cells

Endothelial VGICs have been detected in vivo [54,55,56,109,137,138], and voltage-dependent ion currents have also been measured in freshly isolated endothelial cells [52,54,75,76,78,121]. These observations suggest that VGICs cannot be regarded as an expression artifact caused by long-term in vitro culture [26,104]. The emerging role of VGICs in the vascular endothelium slightly shifts the current view of the signaling role of the endothelial V_M_, which has been primarily associated with endothelium-dependent hyperpolarization [12,18]. Indeed, the activation of VGICs requires membrane depolarization to reach their V_T_, which may vary depending on the corresponding pore-forming α-subunit [1,2]. In order to understand how endothelial VGICs may function, we first need to consider the heterogeneity in the values of the resting V_M_ between large vessels [26,104] and microcirculation, as well as the abundance of depolarizing channels in the vascular endothelium [12,15,140].

### 4.1. The Resting V_M_ of Endothelial Cells Must Shift to Negative Potentials to De-Inactivate VGICs

The average value of endothelial resting V_M_ differs between the macro- and microvasculature. The resting V_M_ of naïve endothelial cells in large vessels was found to be in the range between ≈−60 mV and −40 mV [94,113,114,141], whereas it was between −45 mV and −35 mV in intact resistance arterioles [94,119,142,143]. The resting V_M_ of capillary endothelial cells, which is more challenging to measure in naïve vessels and has therefore been measured in confluent endothelial monolayers, is slightly more depolarized, ranging between −40 mV and −16 mV [52,53,106,144,145]. Therefore, the question arises as to which physiological stimuli lead to VGIC activation in vascular endothelial cells [5,146]. Endothelial Na_V_ (Table 1), Ca_V_ (Table 2), and K_V_ (Table 3) channels are largely inactivated at the resting V_M_. However, vascular endothelial cells may rely on at least two different mechanisms to hyperpolarize and de-inactivate their VGICs: the bistability of resting V_M_ and the expression of multiple hyperpolarizing conductances that can be engaged by physiological cues. Hyperpolarization followed by membrane depolarization represents the most likely bioelectrical signal required to activate VGICs in vascular endothelial cells. In accord, in Table 4 we estimated the fraction of endothelial VGICs available for activation at the resting V_M_ by calculating the steady-state availability (h_∞_) according to the Boltzmann equation when V_M_, V_0.5inact_ and the slope factor for inactivation (k) are known:

$$h_{\infty }=\frac{1}{1+exp(\frac{V_{M}\;-\;V_{0.5\;inact}}{k})}$$

The available values of h_∞_ strongly suggest that the fraction of endothelial VGICs available for activation at the resting V_M_ is remarkably low.

### 4.2. The Resting V_M_ of Endothelial Cells May Be Bistable

Vascular endothelial cells often have a bistable resting V_M_, which may oscillate between ≈−25 mV and ≈−80 mV [80,92,147,148], which is close to the reversal equilibrium for K^+^ (E_K_). The bistability of endothelial V_M_ is primarily driven by the non-linear current–voltage (I-V) relationship of inward-rectifier K_IR_2.1 channels [68,149]. K_IR_2.1 channels do not conduct K^+^ at V_M_ more positive than −40 mV [68,149,150]; such potentials may result by the basal activation of non-selective cation conductances (NSC) and volume-sensitive Cl^-^ channels [53,145,147,151,152], with equilibrium potentials of approximately 0 mV (E_NSC_) and −30/−40 mV (E_Cl_), respectively. However, K_IR_2.1 channels display an interval of negative slope conductance at potentials ranging between E_K_ and −40 mV, which can rapidly clamp the endothelial resting V_M_ at ≈−80 mV following a modest (≤12 mM) increase in extracellular K^+^ concentration ([K^+^]_o_) [144,153,154]. Consistently, it has been estimated that increased activity of excitable cells, such as those in the brain, heart, and skeletal muscle, can lead to an accumulation of K^+^ in the interstitium that can be on the order of 8-10 mM, which is sufficient to activate capillary VGICs [21,149,150]. Interestingly, many endothelial VGICs are located in capillaries (Table 1, Table 2 and Table 3), which are in close proximity to parenchymal cells and are therefore able to function as K^+^ sensors [149,155]. In endothelial cells from large vessels, which are not exposed to such changes in [K^+^]_o_, the shift from positive to negative potentials can be driven by an increase in the activity of the electrogenic Na, K-ATPase (3Na^+^ out: 2K^+^ in) at low extracellular K^+^ concentrations [80,156,157]. The bistability of V_M_ could bring VGICs back from the inactive to the closed state, thereby making them susceptible to activation by an incoming depolarizing stimulus. Additionally, these transitions in the endothelial resting V_M_ could activate either the window current (−60 mV to −30 mV) [90] or the slow tail current [95] through Ca_V_3.1 T-type channels.

### 4.3. Endothelial Cells Express Many Hyperpolarizing and Depolarizing Conductances That Can Favor VGIC Activation in Response to Physiological Stimuli

Vascular endothelial cells respond to a plethora of chemical and physical stimuli that could potentially lead to VGIC activation. Many endothelial agonists have been shown to cause an initial hyperpolarization followed by a sustained depolarization, including acetylcholine, ATP, histamine, bradykinin, thrombin, and H_2_O_2_ [80,115,158,159]. Physiological agonists bind to G_q_PCRs, which activate phospholipase Cβ (PLCβ) to cleave phosphatidylinositol 4,5-bisphosphate (PIP_2_) into the second messengers InsP_3_ and diacylglycerol (DAG) [12]. InsP_3_-induced ER Ca^2+^ release promotes endothelial hyperpolarization by primarily activating small- and intermediate-conductance K_Ca_ channels, namely K_Ca_2.3 (SK_Ca_) and K_Ca_3.1 (IK_Ca_) [12,17,21,160,161]. The following depolarization is sustained by multiple cation channels, including: (1) TRP Canonical 3 (TRPC3) and TRPC6, which are directly gated by DAG [60,61,162]; (2) TRPV4, which is activated by the PLCβ-dependent hydrolysis of PIP_2_ [163]; (3) store-operated channels (SOCs), which are activated by the InsP_3_-dependent drop in the ER Ca^2+^ concentration and are mediated by the interaction of the ER Ca^2+^ sensor STIM1 with either Ca^2+^-selective or non-selective cation channels, such as Orai1 and TRPC1/TRPC4, respectively [20,164]; and (4) TRP Melastatin 4 (TRPM4), which is directly gated by Ca^2+^ released from the ER [165]. We refer the reader to a number of recent reviews that provide comprehensive descriptions of endothelial TRP channels [13,166] and SOCs [20,164]. Unfortunately, the biophysical characterization of agonist-induced changes in V_M_ was mainly carried out when the panel of endothelial depolarizing channels had not yet been identified. Therefore, future investigations are required to dissect the role of these conductances in endothelial depolarization elicited by chemical stimulation. Additionally, laminar shear stress (LSS) has long been known to induce rapid hyperpolarization followed by a sustained depolarization [167,168,169,170]. The rapid hyperpolarization is likely mediated by inwardly rectifying K_IR_2.1 channels, which are sensitive to LSS [171,172]. Furthermore, vascular endothelial cells express the mechanosensitive Piezo1 and TRPV4 channels [173,174], which carry an inward depolarizing current. Therefore, LASS can activate inwardly rectifying K_IR_2.1 channels, thereby producing the initial membrane hyperpolarization [171,172] that can be followed by Piezo1- and/or TRPV4-mediated depolarization.

The mechanisms described above imply that vascular endothelial cells rely on multiple mechanisms to activate VGICs in response to appropriate chemical or mechanical stimulation. A careful review of the literature confirmed that physiological cues do not trigger action potentials in vascular endothelial cells. An early report showed that tetrabutylammonium (TBA), a potent blocker of K^+^ channels, induced action potential-like oscillations in the V_M_ of pig coronary artery endothelial cells. However, pharmacological manipulation revealed that the V_M_ fluctuations were generated in the smooth muscle and then propagated to the vascular endothelium [113].

## 5. Future Perspectives

Widespread evidence now indicates that VGICs are expressed in non-excitable cells [4,5], including vascular endothelial cells (Table 1, Table 2 and Table 3). Furthermore, the innermost layer of blood vessels is endowed with a combination of ion channels that can both de-inactivate VGICs in depolarized cells and shift V_M_ to the V_T_, thereby enabling VGIC activation. Despite these advances, several key issues remain unresolved and must be addressed to fully elucidate the role of VGICs in endothelial ion signaling. First, the in situ expression of VGIC subunits should be systematically investigated by IF in blood vessels isolated from both human samples and animal models of cardiovascular disorders, such as mice and pigs. Second, the endothelial role of many voltage-gated ion currents remains poorly defined (see Table 1, Table 2 and Table 3). Future work should generate transgenic mice with selective endothelial deletion of VGIC subunits, an approach that has already proven effective in defining the function of endothelial N-methyl-D-aspartate (NMDA) receptors [175], TRPM2 [176], TRP Ankyrin 1 (TRPA1) [177], and K_IR_2.1 channels [155]. Third, the biophysical characterization of endothelial voltage-gated ion currents could be performed in naïve vessels by exploiting the primary explant technique developed by the Nilius group [178]. Fourth, endothelial cells display remarkable phenotypic and functional heterogeneity across vascular beds, vessel calibers, and organ-specific microenvironments [179,180]. Therefore, future studies should systematically determine whether the expression, biophysical properties, and physiological roles of VGICs differ between arterial and venous endothelial cells, macrovascular and microvascular districts, and specialized endothelial phenotypes, including cerebral, pulmonary, cardiac, renal, and valvular endothelia. Such investigations will be essential to establish whether observations obtained in a specific endothelial subtype can be generalized across the vascular system or instead reflect context-dependent adaptations. Fifth, VGICs may signal in a flux-independent mode that does not require ion permeation through the selective pore [181,182], as demonstrated for ionotropic glutamate receptors [19,183] and γ-aminobutyric (GABA) receptors [103]. For instance, the voltage-dependent Na_V_1.5 channel may act as an allosteric modulator of the Na^+^/H^+^ exchanger 1 (NHE1) in breast cancer cells, thereby promoting perimembrane acidification, degradation of the extracellular matrix by lysosomal cathepsins, invadopodia formation, and migration [184]. Moreover, excitation–transcription coupling does not necessarily require extracellular Ca^2+^ entry through voltage-dependent Ca_V_1.2 channels. Instead, membrane depolarization could be conveyed to the auxiliary β subunit, which in turn recruits the downstream Ras/ERK/CREB pathway by directly activating the H-Ras exchangers RasGRF1 and RasGRF2 [185]. Taken together, these observations highlight the need to determine whether the Na_V_1.5 and Ca_V_β subunits, as well as other VGIC subunits, also engage flux-independent signaling in vascular endothelial cells.

Confirming that endothelial VGICs play a physiological role in the regulation of cardiovascular and neurovascular function will have a significant impact on endothelial biomedicine. Recent studies have suggested that VGICs expressed in non-excitable cells contribute to non-primary electrical diseases [4,5]. For instance, Ca_V_1.2 channels in vascular smooth muscle cells have been proposed to support atherosclerotic progression [5]; given that Ca_V_1.2 is also expressed in endothelial cells, together with the well-established contribution of the tunica intima to plaque formation and instability [186], this raises the possibility that endothelial Ca_V_1.2 channels contribute to atherosclerotic lesion development. Similarly, a genome-wide association study has identified CACNA1C, the gene encoding Ca_V_1.2, as a susceptibility locus for calcific aortic valve disease (CAVD) [5,187]. CAVD is driven by an active process of fibrocalcific remodeling sustained by valve endothelial cells and fibroblast-like valve interstitial cells [188]. Future work should determine whether Ca_V_1.2 is expressed in valve endothelial cells and whether it contributes to aberrant matrix deposition. Finally, VGICs and their ancillary subunits are frequently up-regulated in tumor cells and contribute to many cancer hallmarks [6,189,190], including angiogenesis. Current evidence indicates that the aberrant expression of the voltage-dependent ion signaling machinery stimulates aberrant vascularization through VEGF secretion. Nevertheless, remodeling of the endothelial transportome also plays a critical role in tumor vascularization in cancer [136,191,192]. Therefore, the aberrant expression and/or function of VGICs in tumor endothelial cells warrants future investigation.

VGICs represent a widespread pharmacological target for the treatment of several cardiovascular and neurological disorders. However, the interpretation of studies investigating endothelial VGICs still requires careful consideration of the pharmacological tools employed, as some modulators may display concentration-dependent off-target effects and limited subtype selectivity. As aforementioned, future studies should therefore combine pharmacological interventions with complementary electrophysiological, molecular, and genetic approaches, including gene silencing and endothelial-specific knockout models, to unequivocally establish the contribution of individual VGIC subtypes to endothelial physiology and pathology. Therefore, the involvement of endothelial VGICs in life-threatening diseases, such as atherosclerosis, CAVD, and cancer, could open unexpected therapeutic perspectives. For instance, several U.S. Food and Drug Administration (FDA)-approved drugs are therapeutically employed to inhibit Ca_V_1.2, including dihydropyridines (e.g., nifedipine, felodipine, and amlodipine), phenylalkylamines (e.g., verapamil), and benzothiazepines (e.g., diltiazem) [42]. The anti-hypertensive drug, nifedipine, has long been known to protect against atherosclerotic progression, but the underlying mechanism remains elusive [193,194]. Future work should assess whether nifedipine targets Ca_V_1.2 channels that are not only expressed in vascular smooth muscle cells but also in endothelial cells. Similarly, a recent investigation showed that Ca_V_1.2 protein was up-regulated in calcified areas of valves obtained from individuals with CAVD compared with valves obtained from individuals without CAVD [195]. Furthermore, chronic administration of verapamil slowed the progression of valve lesion development in a transgenic mouse model of CAVD [195]. The possibility that this attenuation of calcific lesion development is associated with the inhibition of endothelial Ca_V_1.2 channels cannot presently be ruled out. Notably, future investigations should also determine whether the vascular effects of VGIC modulators currently used in experimental settings reflect direct actions on endothelial VGICs or arise, at least in part, from additional off-target mechanisms. The pro-angiogenic role of Na_V_1.5 (see Section 3.1) should also be assessed in tumor-derived endothelial cells. Na_V_1.5 is over-expressed in several types of cancer cells and the pharmacological blockade of Na_V_1.5-mediated *I_Na_* with the anti-epileptic drug ranolazine and phenytoin has been shown to reduce tumour growth, invasion, proliferation and metastasis, although the cardiac side-effects of Nav1.5 inhibition should be taken in account [196,197].

## 6. Conclusions

In conclusion, accumulating evidence indicates that VGICs are expressed in vascular endothelial cells and contribute to the fine-tuning of endothelial signaling despite the absence of classical electrical excitability. Endothelial VGICs, including Na_V_, Ca_V_, and K_V_ channels, appear to participate in key physiological processes such as angiogenesis, vasomotor control, inflammatory responses, and BBB permeability, primarily through the modulation of V_M_ and Ca^2+^-dependent signaling pathways.

The emerging concept that endothelial cells can dynamically regulate their resting V_M_ to enable VGIC activation provides a novel framework to reconcile their non-excitable nature with the presence of voltage-dependent conductances. In this context, the interplay between hyperpolarizing and depolarizing mechanisms may critically determine VGIC availability and function.

Nevertheless, several important questions remain unresolved, including the precise in situ expression profile of VGIC subunits, their biophysical properties in native endothelium, and their contribution to vascular function in vivo. Addressing these issues will require integrating advanced imaging, electrophysiological, and genetic approaches in physiologically relevant models.

Finally, endothelial VGICs may represent previously unrecognized therapeutic targets in non-primary electrical disorders. A deeper understanding of endothelial VGIC signaling could therefore open new avenues for developing targeted strategies in endothelial medicine.

## Figures and Tables

**Table 4:** Fraction of endothelial VGICs available for activation by a depolarizing stimulus at the resting V_M_.

VGIC Subunit	EC Type	V_M_ (mV)	Biophysical Properties	h_∞_ (%)	Ref.
Na_V_1.5	HSVECs	−28 mV	V_0.5act_ ≈ −30 mV and k = 7.2; V_0.5inact_ ≈ −75 mV and k = 5.2	0.012%	[54]
Unidentified Na_V_	RIAECs	−38 mV	V_0.5act_ = −37.5 mV and k = 7.3; V_0.5inact_ = −81.8 mV and k = 8.45	0.56%	[50]
Ca_V_3.1	Rat PMVECs	Ranging between -63 mV and −24 mV	T-type: V_0.5act_ ≈ −28.4 mV and k = 7.084 mV; V_0.5inact_ ≈ −51.4 mV and k = 5.56	87% at −63 mV and 0.7% at −24 mV	[78]
Undefined K_V_	Guinea pig CMECs	−42 mV	K_A_: V_0.5_ act = +7 mV; V_0.5_ inact = −50 mV and k = 13 mV	22.6%	[106]

Abbreviations: CMECs: cardiac microvascular endothelial cells; HSVECs: human saphenous vein endothelial cells; PMVECs: pulmonary microvascular endothelial cells; RIAECs: rat interlobar artery endothelial cells; V_0.5act_ = half-maximum voltage for activation; V_0.5inact_ = half-maximum voltage for inactivation.

## Data Availability

No new data were created or analyzed in this study.
